# Endotracheal Tube Cuff Overinflation Leading to Hypoxic Cardiac Arrest: A Case Report and Review of the Literature

**DOI:** 10.7759/cureus.27610

**Published:** 2022-08-02

**Authors:** Aron Sulovari, Andres Laserna, Stewart Lustik, Sonia Pyne

**Affiliations:** 1 Department of Anesthesiology and Perioperative Medicine, University of Rochester School of Medicine and Dentistry, Rochester, USA

**Keywords:** ambulatory anesthesiology, cardiac arrest, hypoxic respiratory failure, endotracheal tube overinflation, intraoperative complications, endotracheal tube obstruction

## Abstract

Endotracheal tube cuff overinflation has been shown to produce airway obstruction and subsequent ventilatory and hemodynamic compromise. Although rare, this complication is reversible and its prompt identification is paramount. We describe a case of a 68-year-old woman undergoing microlaryngoscopy and vocal cord lesion biopsy, who developed ventilatory failure and cardiac arrest following endotracheal tube overinflation intraoperatively. The patient was successfully resuscitated and was able to be ventilated after endotracheal tube replacement. We present a literature review and evidence-based management insights for endotracheal tube obstruction due to cuff overinflation.

## Introduction

Endotracheal tube (ETT) cuff overinflation has been shown to cause ventilatory complications in case reports dating as early as 1957 [1,2]. Herniation of the ETT cuff’s plastic lining into the lumen or around the end of the tube results in airway obstruction. In addition, an overinflated cuff can exert increased tracheal pressure, leading to restriction of mucosal blood flow resulting in ischemic complications. Experimentally it has been determined that cuff to tracheal wall pressures exceeding 50 cm H_2_O completely block tracheal mucosa blood flow [3]. Obstructed mucosal blood flow occurs because even when inflated at the recommended intracuff pressure, the cuff exerts approximately 30 cm H_2_O of pressure on the tracheal wall [4]. Nitrous oxide may be associated with an increased likelihood of herniation due to gas diffusion into the closed space, leading to expansion [5]. Diffusion rates into cuffs were inversely proportional to cuff thickness and directly proportional to the partial pressure of nitrous oxide. Hyperinflated cuffs may also incur damage to the intralaryngeal portion of the recurrent laryngeal nerve and cause post-extubation hoarseness [6].

In this manuscript, we present a case of a 68-year-old woman undergoing microlaryngoscopy at an ambulatory surgical center (ASC) who intraoperatively developed sudden ventilatory and hemodynamic compromise secondary to ETT cuff overinflation and suspected airway obstruction. Additionally, we performed a literature review to increase awareness of this possible complication.

## Case presentation

A 68-year-old woman with a left supraglottic lesion of undetermined significance presented to an ASC for a microlaryngoscopy and biopsy with use of a carbon dioxide (CO_2_) laser. The patient was referred to otolaryngology for management of voice change and sore throat following an esophagogastroduodenoscopy (EGD). A CT scan of the neck was negative and a flexible laryngoscopy revealed a left supraglottic lesion with surrounding erythema (Table 1). She was a former smoker with a 40-pack-year tobacco history, asthma treated with budesonide/formoterol and albuterol as needed, hypertension well controlled with carvedilol, and bilateral carotid artery stenosis status post carotid endarterectomy and stent placement for which she was on clopidogrel daily. The physical exam was unremarkable except for distant breath sounds noted bilaterally. She previously underwent EGD and carotid endarterectomy with stent placement under deep sedation without complication. The patient was 152 cm in height, and 57.5 kg with a BMI of 24.9 kg/m^2^. She had a previous anaphylactic reaction to amoxicillin/clavulanate and sensitivities to statins, celecoxib, and buproprion.

**Table 1:** Results of flexible laryngoscopy and biopsy

Flexible Fiberoptic Laryngoscopy
Pre-Procedure Diagnosis: throat pain, voice change
Post-Procedure Diagnosis: supraglottic lesion
Findings:
Supraglottic hyperfunction: none
Right Vocal Fold Movement:
Abduction: normal
Adduction: normal
Longitudinal tension (cricothyroid): normal
Left Vocal Fold Movement:
Abduction: normal
Adduction: normal
Longitudinal tension (cricothyroid): normal
Arytenoid Joint Movement: normal
Arytenoid Mucosa: normal
True Vocal Fold Characterstics: normal
Mass(es)/Vibratory Margin Irregularites: none
Other Structural Lesions: left false vocal fold with erosion of the anterior mucosa extending to the petiole of the epiglottis
Surgical Pathology
FINAL DIAGNOSIS:
Larynx, left supraglottis, biopsy:
- Papillary squamous lesion. See comment.
Comment: Specimen is predominantly superficial epithelium with papillary features. Scant submucosal tissue is present for evaluation. If there is concern for a more serious process, further diagnostic studies are recommended.

The patient was premedicated with 1 mg of midazolam and 50 mcg of fentanyl, induced with 100 mg propofol and 30 mg of rocuronium, and intubated routinely with a 6.0 specialized ETT using direct laryngoscopy with a Macintosh 3 blade. A Covidien Shiley Laser-Flex Dual Cuffed Tracheal Tube specifically designed for airway surgeries involving laser technology was chosen for the procedure. Both the tracheal cuff and the proximal cuff were inflated with 5 mL saline at the time of intubation without incident. The patient tolerated a general anesthetic maintained with a mixture of sevoflurane, air, and oxygen (21%-33% FIO_2_ during laser use) until approximately 40 minutes into the procedure. At this point, while performing the microlaryngoscopy, the surgeon repeatedly expressed concern about a potential air leak around the ETT. The surgeon reinflated the ETT cuff with additional saline, approximately three more times with an additional 5-8 mL of saline each time It was noted quickly thereafter that there was a sudden loss of end-tidal CO_2_ and breath sounds bilaterally could not be auscultated. Severe bronchospasm was suspected and the patient was given albuterol via the ETT as well as 10 mcg of epinephrine intravenously (IV) with no effect. The ETT was suctioned with a suction catheter without resistance and improvement in the patient’s condition. On EKG monitoring, the patient’s heart rate initially dropped to 30 bpm and 1 mg of atropine was administered with no effect. Within seconds, her pulse was nonpalpable and the EKG revealed asystole. The patient was still unable to be ventilated. A code was called. 1 mg of epinephrine was administered IV as chest compressions were initiated. The Laser-Flex ETT was removed and replaced with a standard 7.5 oral ETT which resulted in bilateral breath sounds and a normal end-tidal CO_2_ tracing and palpable radial pulse. An arterial line was placed for closer monitoring as well as a second large bore IV. The immediate transfer was arranged for the patient by ambulance from the ASC to the emergency department of the affiliated university hospital.

## Discussion

We screened Medline and Embase using the following search strategy: (“endotracheal tube obstruction” OR “obstructed endotracheal tube” OR “tracheal tube obstruction” OR “endotracheal tube overinflation” OR “intracuff pressure”) AND (“anesthesia” OR “anesthesiology” OR “intraoperative” OR “surgery” OR “operative”). We screened 1,651 studies using the artificial intelligence software Abstrackr® (Brown University, School of Public Health, Providence, USA) [7].

In this case report and literature review, we discuss that overinflation of ETT cuffs may result in cuff pressures that may obstruct the tube and if ventilation is not reestablished quickly, hemodynamic instability and hypoxia may ensue. Herein a case of ETT overinflation resulting in hemodynamic and ventilatory compromise is presented. A 68-year-old woman undergoing microlaryngoscopy developed hypoxic cardiac arrest after cuff reinflation intraoperatively. Advanced cardiac life support (ACLS) was initiated and replacement of the ETT resulted in the return of ventilation and spontaneous circulation. It is reasonable to conclude that airway compromise occurred due to manual cuff overinflation. To the best of our knowledge, this is one of the few case reports demonstrating airway obstruction secondary to ETT cuff overinflation occurring intraoperatively in an ASC setting.

ETT obstruction does not appear in recent algorithms for the management of ventilation failure in intubated patients. Recent guidelines recommend optimization of oxygenation, evaluating whether to restore spontaneous breathing, and considering a noninvasive vs. invasive device for emergent and unanticipated difficult airway management [8]. Although direct visualization via bronchoscopy would clearly demonstrate the obstruction, bronchoscopy is not emergently available in every setting. An unclear etiology may lead to dangerous extubation and reintubation. Accordingly, Zenga et al have suggested a trial of cuff deflation in intubated patients with new onset ventilation difficulty [9]. In our case replacement of the ETT tube achieved a similar effect.

Risk factors contributing to major complications associated with airway management must be identified, including but not limited to poor airway assessment, multiple intubation attempts, obesity, and anesthesia for head and neck surgery such as in this case [10]. Excessive air in the ETT cuff should be avoided. In a mechanically ventilated patient, obstruction of the airway should be suspected by high peak inspiratory pressures and decreased lung compliance, which may progress to a decrease in oxygen saturation and an abnormal CO_2_ tracing and vital signs. A sudden increase in inspiratory pressures could be due to multiple other factors in addition to ETT obstruction, including reactive airways, tension pneumothorax, mainstem intubation, or malfunction of the ventilator or breathing circuit. If there are significant ECG and hemodynamic changes as in the case presented, ACLS should be initiated. It is important to identify if there were any recent intraoperative adjustments to ETT cuff pressure or tube position. If so the ETT position should be assessed and adjusted accordingly. If the ETT is appropriately placed a trial of cuff deflation is warranted to relieve an ETT obstruction. In lieu of changes in ETT position or cuff pressure, the obstruction may also be assessed by passing an appropriate size suction catheter to inspect patency, although this has not always been reliable, including in our case [11]. If these measures fail, additional etiologies should be considered.

Regular monitoring of intracuff pressures and routine deflation and reinflation of the cuff to the no-leak point may prevent cuff overinflation. However, identifying an optimal method of doing so is challenging and poorly studied. Methods such as manual palpation, which is commonly recommended, is an unreliable methods for estimating cuff pressure [12]. There are several commercially available cuff inflator measuring devices available to assist in this problem [13]. A recent randomized control trial found that automated vs. manual correction of cuff pressure was associated with a significantly lower rate of underinflation (2% vs. 15%, P<0.001). However, there were no significant differences in overinflation rates (2% for both, P=0.78) [14]. In their editorial highlighting the importance of monitoring ETT intracuff pressure in ICU and anesthesia patients, Kumar et al. recommend continuous intracuff pressure monitoring [15]. However given the lack of high-quality data to support the routine use of monitoring technology, the authors do not recommend potentially costly continuous intracuff pressure monitoring technology.

Anesthesiologists should also be aware of common risk factors for cuff overinflation, namely the use of N_2_O, tube positioning, and length of the procedure. Combes et al. conducted a study of 50 patients where the ETT cuff was inflated with either saline or air. The authors found that excess cuff pressure during balanced anesthesia with isoflurane and nitrous oxide resulted in a greater incidence of a sore throat for the air treatment group in the postanesthesia care unit (76% vs 20%) and 24 hours after extubation (42 vs. 12%, P<0.05 for both). Tracheal lesions at the time of extubation, as assessed by fiberoptic examination, were seen in 100% of patients in the air group versus 32% of patients in the saline group. These results indicate that N2O diffusion in the cuff inflated with air may result in tracheal mucosal erosion and sore throat perioperatively [16]. Hyperextension of the neck has also been shown to increase ETT cuff pressure [17]. This is an important consideration in surgeries that require fixation of the head such as ear, nose, and throat, and neurosurgeries. There can also be significant variations in intracuff pressure during prolonged surgical procedures of more than four hours [18].

We reviewed all case reports in which cuff overinflation caused significant respiratory or hemodynamic complications. We reviewed 56 full-text manuscripts and included four case reports (Table 2).

**Table 2:** Review of the literature

Author	Year	Country	Case summary (age, procedure, ETT used )	Clinical Presentation	Outcome	Recommendations
Perel et al. [19].	1977	USA	Case 1: 55-year-old man undergoing craniotomy, remained intubated postoperatively with high compliance Warne nasotracheal tube, 8.5 mm internal diameter (ID). Case 2: 14-year-old female undergoing mitral and aortic valve replacement, remained intubated postoperatively with high compliance Warne nasotracheal tube 7 mm ID.	Case 1: Increase in peak inspiratory pressure without change in tidal volume, inability to pass suction catheter. Case 2: Tachypnea and reduced breath sounds.	Resolution of tachypnea and ventilatory difficulties by deflation and reinflation of cuff.	Regular monitoring of intracuff pressures, inflating the cuff with N_2_0-0_2_, routine deflation and reinflation of the cuff to the no-leak point, and the use of a pressure-relief valve.
Johnson and Lehman [13].	2012	USA	13-year-old male who sustained a closed head injury and was intubated with a 6.0 mm ID endotracheal tube (ETT) in the field	Diminished breath sounds and chest rise, inability to pass suction catheter, end tidal CO_2_ of 70 mmHg.	Cuff deflation improved respiratory status.	Frequent measurement and adjustment of the ETT cuff pressure, possibly with the assist of monitoring devices.
Gleich et al. [5].	2008	USA	56-year-old man undergoing total thyroidectomy and was intubated with a 7.0 mm ID electromyographic ETT.	Increase of peak inspiratory pressure from 28 to 50 mmHg, difficulty in manual ventilation, spO2 of 90%.	Cuff deflation restored adequate ventilation.	Continuous monitoring of intracuff pressure, although rarely used in routine clinical practice, could have prevented this complication.
Hofstetter et a.l [20].	2010	Germany	8-year-old boy admitted to emergency following traffic accident intubated with 6.0 mm ID ETT.	Decreased breath sounds, increase in peak inspiratory pressure and inability to pass a suction catheter.	ETT removal and reintubation restored baseline ventilation parameters.	A manufacturer lead trial to demonstrate obstruction of the ETT by its cuff.

## Conclusions

ETT cuff overinflation leading to ETT obstruction is a dangerous complication that can present during anesthesia due to difficulties in continuous monitoring of cuff pressures. Rapid identification of this presentation is crucial in preventing critical hypoxia and hemodynamic compromise. This complication is readily reversible with proper identification as illustrated by the literature review and our case report.
